# The JNK pathway represents a novel target in the treatment of rheumatoid arthritis through the suppression of MMP-3

**DOI:** 10.1186/s13018-020-01595-9

**Published:** 2020-07-17

**Authors:** Tomotake Kanai, Naoki Kondo, Masayasu Okada, Hiroshige Sano, Go Okumura, Yasufumi Kijima, Akira Ogose, Hiroyuki Kawashima, Naoto Endo

**Affiliations:** 1 Division of Orthopedic Surgery, Department of Regenerative and Transplant Medicine, Niigata University Graduate School of Medical and Dental Sciences, Chuo-ku, Niigata, Niigata Japan; 2 Department of Neurosurgery, Brain Research Institute, Niigata University, Chuo-ku, Niigata, Niigata Japan; 3 Department of Orthopedic Surgery, Uonuma Institute of Community Medicine, Niigata University Medical and Dental Hospital, Minami-Uonuma, Niigata, Japan

**Keywords:** Low-dose NAC, MMP-3, JNK pathway, MH7A, RA-FLS

## Abstract

**Background and aim:**

The pathophysiology of rheumatoid arthritis (RA) is characterized by excess production of pro-inflammatory cytokines, including tumor necrosis factor-α (TNF-α), interleukin-1β (IL-1β), and interleukin-6 (IL-6) by neutrophils and macrophages in synovium. Additionally, these cytokines promote the production of reactive oxygen species (ROS), and increased production of matrix metalloproteinases (MMPs), including MMP-3, in synoviocytes that result in joint destruction. There is limited information on how proteolytic enzymes such as MMP-3 can be regulated. We evaluated the effect of the antioxidant *N*-acetylcysteine (NAC) on RA and identified the relationship between the c-Jun N terminal kinase (JNK) pathway and MMP-3. We hypothesized that elucidating this relationship would lead to novel therapeutic approaches to RA treatment and management.

**Methods:**

We investigated the effect of administering a low dose (1000 μM or less) of an antioxidant (NAC) to human rheumatoid fibroblast-like synoviocytes (MH7A cells). We also investigated the response of antioxidant genes such as nuclear factor erythroid -derived 2-related factor 2 (Nrf2) and Sequestosome 1 (p62). The influence of MMP-3 expression on the JNK pathway leading to joint destruction and the mechanisms underlying this relationship were investigated through primary dispersion culture cells collected from the synovial membranes of RA patients, consisting of rheumatoid arthritis-fibroblast-like synoviocytes (RA-FLS).

**Results:**

Low-dose NAC (1000 μM) increased the expression of Nrf2 and phospho-p62 in MH7A cells, activating antioxidant genes, suppressing the expression of MMP-3, and inhibiting the phosphorylation of JNK. ROS, MMP-3 expression, and IL-6 was suppressed by administering 30 μM of SP600125 (a JNK inhibitor) in MH7A cells. Furthermore, the administration of SP600125 (30 μM) to RA-FLS suppressed MMP-3.

**Conclusions:**

We demonstrated the existence of an MMP-3 suppression mechanism that utilizes the JNK pathway in RA-FLS. We consider that the JNK pathway could be a target for future RA therapies.

## Background

Studies on the treatment of rheumatoid arthritis (RA) have focused on biological agents that suppress pro-inflammatory cytokines, including TNFα, IL-1β, and IL-6 [1–3]. However, the suppression of oxidative stress, which causes inflammation, has not been sufficiently investigated [3]. Antioxidants, including *N*-acetylcysteine (NAC) that eliminate reactive oxidative species (ROS), are used to suppress oxidative stress [4–6]. NAC directly suppresses and eliminates ROS [4, 7]. ROS promotes the production of matrix metalloproteinase- (MMP-) 3, a proteolytic enzyme that induces joint destruction and suppressing ROS might suppress MMP-3 [1–4, 8, 9].

In human synoviocytes stimulated with TNFα, high dose of NAC (30 mM) administration suppressed nuclear factor-kappa B (NF-κB ) activation and production of TNFα and IL-6 proteins [10]. High doses of NAC have an anti-inflammatory effect; however, Sadowska et al. indicated that doses of 10 mM and higher are cytotoxic. Zafarrullah et al. found that low doses of NAC (0.1–1 mM) regulated the redox state but doses higher than 10 mM resulted in structural alterations of TGF-β [4, 11]. In a clinical setting, caution is required when determining the appropriate NAC dose.

The mitogen-activated kinase (MAPK) signal transduction pathway is associated with ROS activity and a p38 inhibitor suppressed ROS in HeLa cells treated with H_2_O_2_ [12]. JNK pathway stimulates inflammatory activity. It was prominently suppressed by administering NAC after hepatic ischemia-reperfusion injury in mice [7]. NAC treatment eliminates ROS and suppresses the JNK pathway and thereby protects granulosa cells from H_2_O_2_-induced apoptosis [13]. NAC affects the activity of MAPKs (mainly JNK); however, human synoviocytes display large individual differences in the expression of interleukins and MMPs. Owing to these differences, human synoviocytes were considered unsuitable for clarifying the mechanisms, including signal transduction and influence of the JNK pathway. For this reason, experiments were carried out on cell lines with less individual variation in the expression of interleukins and MMPs, particularly MH7A cells, which are human fibroblast-like synoviocytes. Our primary objective was to investigate the effect of low-dose NAC on RA. To achieve this, we attempted to confirm that MMP-3 expression is linked to anti-oxidative effects, anti-inflammatory activity, and joint destruction and determine its underlying MAPK signal transduction pathway. Our ultimate goal was to utilize the knowledge of signal transduction pathways to establish novel RA therapies using specific inhibitors.

## Methods

### Cell culture and chemicals

The MH7A rheumatoid fibroblast-like synoviocyte cell line was obtained from the Institute of Physical and Chemical Research (RIKEN, Tsukuba, Ibaraki, Japan). The pelleted MH7A cells were stored at − 80 °C and cultivated in RPMI 1640 medium (Gibco, Grand Island, NY, USA) supplemented with 10% fetal bovine serum (FBS) (Biowest, Nuaillé, France) and 1% antibiotic/antimycotic solution (Invitrogen, Carlsbad, CA, USA) in a humidified incubator with 95% air and 5% CO_2_ at 37 °C. After the fifth passage, MH7A cells were seeded into 3.5 cm dishes at a concentration of 3 × 10^5^ cells/well and cultured for three days until 80–90% confluency was achieved. These cells were then examined.

Synovial tissues were obtained from two RA patients undergoing total knee arthroplasty and one patient undergoing synovectomy of the wrist. These patients had been diagnosed with RA according to the revised criteria of the American College of rheumatology [14] and had been treated with biologics, methotrexate, prednisolone, immunosuppressants, disease-modifying anti-rheumatic drugs (DMARDs), and nonsteroidal anti-inflammatory drugs (NSAIDs) (Table 1).

**Table 1:** Details of RA patients who collected synoviocytes

	Sex	Age	Drug (pre-operation)	CRP (mg/dl) (pre-operation)	ESR (mm/1 h) (pre-operation)
FLS 1	Female	43	Methotrexate 10 mg/week Prednisolone 5 mg/week Bredinin 450 mg/day NSAIDs	0.09	10
FLS 2	Female	56	Adalimumab 40 mg/2 week Methotrexate 8 mg/day NSAIDs	0.21	5
FLS 3	Female	85	Iguratimod 50 mg/day Bucillamine 100 mg/day	1.54	91

*CRP* C reactive protein, *ESR* erythrocyte sedimentation rate

Written informed consent was obtained from each patient before the specimens were collected in accordance with the protocols of the Niigata University Medical and Dental Hospital ethics committee. RA-FLS were isolated using the methods of Rosengren et al. [15] and Sano et al. [16]. Briefly, synovial tissues were cut into small pieces and digested with RPMI 1640 medium mixing collagenase (1 mg/mL) (Worthington Biochemical Corporation, Lakewood, NJ, USA) for 3 h. The tissue was then filtered using a 70 μM nylon cell strainer, washed, and suspended in RPMI 1640 medium. Dissociated cells were then centrifuged at 1500×*g* for 3 min twice and re-suspended in RPMI 1640 medium supplemented with 10% FBS and 1% antibiotic/antimycotic solution. Cells were cultured overnight, the non-adherent cells were removed, and the adherent cells were cultivated in RPMI 1640 medium supplemented with 10% FBS and 1% antibiotic/antimycotic solution. After the fifth passage, RA-FLS were seeded into 3.5 cm dishes at a concentration of 3 × 10^5^ cells/well and cultured for 3 days until 80–90% confluency was achieved. These cells were then examined.

NAC (Sigma-Aldrich, St. Louis, USA), a specific JNK inhibitor, SP600125 (Sigma-Aldrich), H_2_O_2_ (WAKO, Osaka, Japan), and dimethyl sulfoxide (DMSO; Meso Scale Discovery, Rockville, MD, USA) were used. Primary antibodies specifically recognizing IL-6 (Cell Signaling Technology, Danvers, MA, USA), MMP-3 (Cell Signaling Technology), Nrf2 (Abcam, Cambridge, UK), β-actin (Sigma-Aldrich), and phosphorylated antibody specifically recognizing phosphorylated forms of p62 (MBL, Nagoya, Japan), and JNK (Cell Signaling Technology) were also used.

### Evaluation of cell viability

The effect of NAC on cell viability was determined using the XTT assay (Cell Proliferation Kit II, Roche Diagnostics, Basel, Switzerland), which is based on the reduction of a tetrazolium salt by mitochondrial dehydrogenase in viable cells. Cells were seeded into a 96-well plate at a density of 5 × 10^4^ cells/mL and treated with different concentrations of NAC ranging from 10 μM to 10 mM for 24 h at 37 °C in 5% CO_2_. Then, 50 μL of XTT stock solution (0.3 mg/mL) was added to each well to attain a total volume of 150 μL. After incubation for 18 h, the optical density (OD) _450-500_ was read on a scanning multi-well spectrophotometer (Model 680, Bio-Rad, Hercules, CA, USA).

### Western blotting

MH7A cells in 3.5 cm dishes were incubated with medium containing NAC or SP600125 for 3 and 24 h. RA-FLS in 3.5 cm dishes were incubated with medium containing SP600125 for 3 and 24 h. Treated cells were washed with phosphate-buffered saline (PBS) (non-Ca and Mg) and harvested with a cell scraper. To prepare whole cell lysates, cell pellets were extracted with lysis buffer containing 1× Laemmli/urea (62.5 mM Tris, pH 6.8, 2% sodium dodecyl sulfate, 5% glycerol, and 6 M urea) and proteinase inhibitor (4 μL). After measuring the protein concentration in the supernatant using the Pierce^TM^ BCA Protein Assay Kit (Thermo Fisher Scientific, Waltham, MA, USA), the supernatants were mixed with 5% (v/v) 1 M dithiothreitol and 5% (v/v) bromophenol blue and heated at 98 °C for 5 min. Equal amounts (50 μg/lane) of proteins were separated by 10% sodium dodecyl sulfate-polyacrylamide gel electrophoresis (SDS-PAGE) and then electro-transferred onto nitrocellulose membranes. The membranes were incubated with the indicated primary antibodies (IL-6, MMP-3, Nrf2, phosphorylated p62, phosphorylated JNK) and further incubated with secondary G-horseradish peroxidase conjugates (Amersham^TM^, GE Healthcare, Little Chalfont, UK). Protein bands were visualized by Western blotting detection solution using an enhanced chemiluminescence Western blotting detection solution (Hi-RENDOL®, Hi-RENFIX®, Fujifilm, Tokyo, Japan) and exposing the membranes to X-ray film or the protein signals were detected with an ECL system (BioRad, Hercules, CA, USA) and visualized using a charge-coupled device (CCD) cooled camera (Gene Genome; Syngene, Cambrigde, UK). These X-ray film data were digitalized and graphs were individually created using public domain image processing software, ImageJ (U. S. National Institutes of Health, Bethesda, Maryland, USA, http://imagej.nih.gov/ij/).

### Immunocytochemistry/immunofluorescence and visualization of Nrf2

MH7A cells were cultured in chamber slides (SUPERFROST®, Matsunami Glass, Japan) for 24 h following pre-incubation for 3 and 24 h after the administering of NAC (1000 μM). Cells were again cultured for 1 h after the administering of H_2_O_2_ (100 μM). The cells were then fixed with 4% paraformaldehyde and permeabilized with Tris-buffered saline (TBS) containing 0.1% Triton X-100. Nonspecific binding was blocked with 5% bovine serum albumin (BSA) dissolved in Dulbecco’s phosphate-buffered saline (DPBS) for 30 min. The slides were incubated for 1 h at 25 °C with primary antibody (anti-Nrf2, ab53019, Abcam). After three washes for 10 min each, the slides were incubated for 1 h with Alexa Fluor™ 568 Phalloidin supplemented with goat anti-rabbit IgG- Alexa Fluor 488(Thermo Fisher Scientific) secondary antibodies. Nuclei were stained with 4′,6-diamidino-2-phenylindole (DAPI; Thermo Fisher Scientific). After three washes for 10 min each, the slides were covered with mounting medium (Dako, Glostrup, Denmark) and analyzed with a confocal laser scanning microscope (FLUOVIEW 1200, Olympus, Tokyo, Japan).

### Evaluation of ROS formation

Quantitative ROS measurements for MH7A cells incubated in medium containing NAC or SP600125 for 3 and 24 h was performed using the Muse™ Oxidative Stress kit (EMD Millipore Bioscience, Billerica, MA, USA). This provided the relative percentages of cells that are ROS-negative and ROS-positive. After treatment with NAC or SP600125, MH7A cells were harvested, incubated with oxidative stress reagent (dihydroethidium), and analyzed on the Muse Cell Analyzer according to the manufacturer’s protocol. To measure ROS, a reflection of oxidative stress, MH7A cells were incubated in medium containing NAC or SP600125 for 3 and 24 h each, and H_2_O_2_ was added to the medium for 1 h because the intracellular ROS level was highest 1 h after the addition of H_2_O_2_ [17, 18].

### Chemiluminescent enzyme immunoassay

The concentrations of IL-6 in the MH7A cell culture supernatants were measured using a Fully Automated Chemiluminescent Enzyme Immunoassay system (LUMIPULSE® G1200, Fujirebio, Inc., Tokyo, Japan). IL-6 in MH7A cells was measured after administering NAC (1000 μM) for 24 h or SP600125 (30 μM) following 24 h of treatment with 100 μM H_2_O_2_ to induce oxidative stress.

### Statistical analysis

All measurements were replicated three or four times, and all values are expressed as the means ± the standard error of the mean (SEM). Statistical analyses were performed with one-way analysis of variance to analyze (ANOVA) followed by Turkey’s multiple comparisons test, and two-way ANOVA followed by Dunnett’s, Turkey’s, or Bonferroni’s multiple comparisons test using GraphPad Prism software (GraphPad, Inc., San Diego, CA, USA). *P* value < .05 was considered statistically significance.

## Results

### Determination of working concentration of NAC and H_2_O_2_ in MH7A cells

**Fig. 1:**
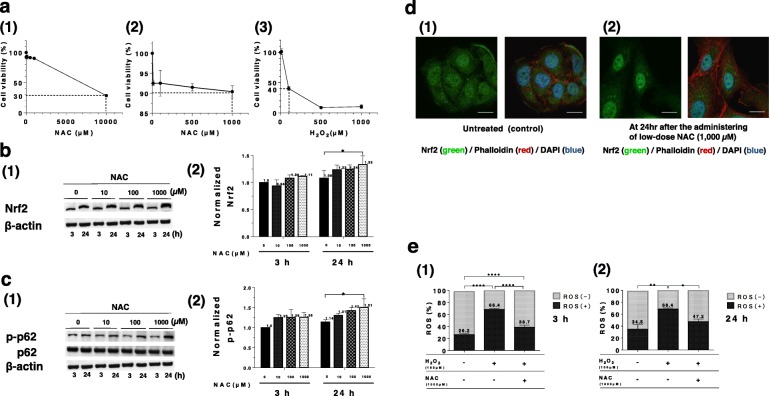
Anti-oxidative effects of NAC. **a**(1) Cell viability graphs following NAC treatment at concentrations of 10, 100, 500, 1000 μM, and 10 mM for 24 h. All data are expressed as the mean ± SEM (*N* = 4). The dash-dotted line is a 10 mM NAC concentration producing 30% cell viability. **a**(2) This graph is an enlargement of **a**(1) below 1000 μM. The concentration of NAC showing less than 10% cytotoxicity was 1000 μM. **a**(3) The cell viability assay of H_2_O_2._ H_2_O_2_ more than 100 μM was cytotoxic, killing 60% of cells in the sample. **b**(1), **c**(1) An illustrative Western blotting picture of Nrf2 and phospho-p62 (p-p62) following NAC treatment (1000 μM) for 3 or 24 h. **b**(2), **c**(2) Normalized values of Nrf2 or p-p62 were graphed (*N* = 3). Normalization was performed with Nrf2 or p-p62 band intensity of untreated cell (3-h incubation). **d** Immunocytochemistry of MH7A cells stained to identify Nrf2. The color analyzed by confocal laser scanning microscopy is expressed as Nrf2 (green)/phalloidin (red)/DAPI (blue). **d**(1) Untreated cells were cultured for 24 h. **d**(2) Cells cultured for 24 h treated with low-dose NAC (1000 μM). Scale bar; 20 μm. **e** ROS formation in MH7A cells following H_2_O_2_ treatment (100 μM) for 1 h and low-dose NAC treatment (1000 μM) for 3 (**e**(1)) and 24 h (**e**(2)). All values are expressed as the mean ± SEM (*N* = 3). The significance levels are shown as *P value < .05, **P value < .01, ***P value < .001, ****P value < .0001

To determine the appropriate experimental concentrations of NAC and H_2_O_2_ for the experiment, we determined the cytotoxicity of these solutions. At a concentration of 10 mM (10,000 μM) NAC, cell viability of MH7A was 30% after 24 h of treatment (Fig. 1a(1)). At a concentration of 1000 μM or lower NAC, cell viability of MH7A was greater than 90%. We, therefore, chose 1000 μM or lower NAC as our working concentration (Fig. 1.a(2)). On the other hand, H_2_O_2_ is typically used at concentrations from 100 to 1500 μM [13, 17, 18]. MH7A cell lived about only 40% by administering 100 μM of H_2_O_2_ for 24 h. We decided to use 100 μM of H_2_O_2_ for a brief time (Fig. 1a(3)).

### Low-dose NAC increases Nrf2 and p62 (antioxidant-related proteins) and suppresses ROS elevation following H_2_O_2_ treatment

Nrf2 protein expression showed dose- and time-dependent increases at 3 and 24 h after the administration of NAC (10–1000 μM). Its expression significantly increased at 24 h after administering NAC (1000 μM) compared with that at 24 h without treatment of NAC (1.33 vs. 1.08, *P* = .036) (Fig. 1b(1, 2)). Phosphorylation of p62 (serine 403-phosphorylated p62) also showed dose- and time-dependent increases at 3 and 24 h after the administration of NAC (10–1000 μM). Its expression significantly increased at 24 h after administering NAC (1000 μM) compared with that at 24 h without treatment of NAC (1.14 vs. 1.51, *P* = .037) (Fig. 1c(1, 2)).

Nrf2 expression was higher in cytoplasm than in nucleus after 24 h in untreated MH7A cells (Fig. 1d(1)). At 24 h after administering NAC (1000 μM), Nrf2 was translocated from cytoplasm to nucleus reflecting antioxidant response (Fig. 1d(2)). This phenomenon is known to be always caused by oxidative stress.

The mean positive percentage of ROS in MH7A cells was 26.2% at 3 h, and 34.5% at 24 h, without NAC treatment. Compared with these control data, there was a significant increase in ROS (68.4%) 1 h after the administering H_2_O_2_ (100 μM) (*P* < .0001). Pre-incubation with NAC (1000 μM) for 3 and 24 h followed by 1 h of H_2_O_2_ (100 μM) treatment resulted in significant decreases in ROS levels from 68.4 to 38.7% at 3 h (*P* < .0001), and from 68.4 to 47.2% at 24 h (*P* = .019), respectively (Fig. 1e(1, 2)). These results demonstrated that low-dose NAC (1000 μM) suppressed the ROS elevation following H_2_O_2_ treatment (100 μM) in MH7A cells.

### Low-dose NAC suppresses MMP-3 protein expression and phosphorylation of JNK in MH7A cells

**Fig. 2:**
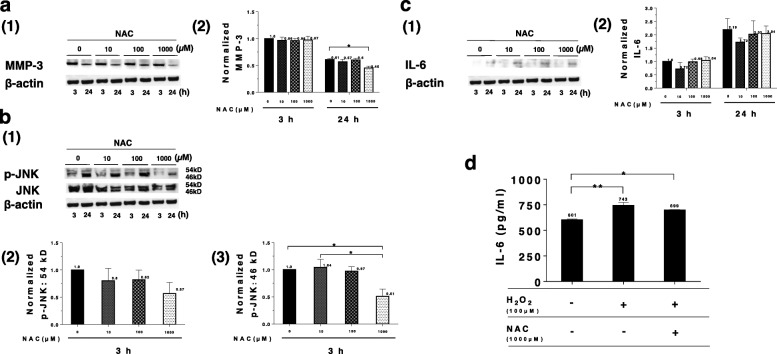
Low-dose NAC suppressed MMP-3 expression and JNK phosphorylation. **a**(1) An illustrative Western blot picture of MMP-3 following low-dose NAC treatment (1000 μM) after 3 and 24 h. **a**(2) Normalized intensities were graphed (*N* = 3). Normalization was performed with MMP-3 band intensity of untreated MH7A cell (3-h incubation). **b**(1) An illustrative Western blot picture of phosphorylated JNK (54 and 46 kD) in MH7A cells following NAC (10, 100, and 1000 μM) for 3 and 24 h. **b**(2, 3) Normalized intensities were graphed (*N* = 3). Normalization was performed with phosphorylated JNK (p-JNK; 54 and 46 kD) of untreated MH7A cell (3-h incubation). **c**(1) An illustrative Western blot picture of IL-6 in MH7A cells following low-dose NAC treatment (1000 μM) after 3 or 24 h. **c**(2) Normalized intensities were graphed (*N* = 3). Normalization was performed with IL-6 bad intensity of untreated MH7A cell (3-h incubation). **d** This graph showed the mean concentrations of IL-6 in supernatant of MH7A cells determined by chemiluminescent enzyme immunoassay (CLEIA) following 24 h after H_2_O_2_ treatment (100 μM) and low-dose NAC treatment (1000 μM). All values are expressed as the mean ± SEM (*N* = 3). The significance levels are shown as *P value < .05, **P value < .01

Expression of MMP-3 protein was not altered 3 h after administering NAC (10, 100, and 1000 μM); however, it decreased significantly after 24 h of NAC administration (1000 μM) compared with that without NAC treatment (0.45 vs. 0.61 in the band expression intensity, *P* = .03) (Fig. 2a(1, 2)). Phosphorylation of JNK (54 kD) did not demonstrate significant dose-dependent decrease at 3 h following low-dose NAC treatment. However, phosphorylation of JNK (46 kD) decreased significantly 3 h after NAC administration (1000 μM) compared with those at 3 h without NAC treatment and 10 μM NAC treatment (1.0 vs. 0.51in the band expression intensity, *P* = .049, and 1.04 vs. 0.51, *P* = 0.033, respectively) (Fig. 2b(1, 2, 3)).

To investigate the change of IL-6 expression under treatment of NAC in MH7A cells, we performed the same experiments as MMP-3 and JNK proteins. Expression of IL-6 protein was not significantly changed at 3 and 24 h after the administering of NAC (10, 100, and 1000 μM) compared with the condition without NAC treatment (Fig. 2c(1, 2)).

H_2_O_2_ (100 μM) administration for 24 h significantly increased IL-6 concentration in supernatant of MH-7A cells compared with untreated condition (743 vs. 601 pg/ml, *P* = .0047). NAC treatment (1000 μM) for 24 h slightly decreased IL-6 concentration compared with the condition under H_2_O_2_ (100 μM) but no significant difference was detected (699 vs. 743 pg/ml). These findings indicated that NAC was not able to reduce IL-6 expression in cell lysates or IL-6 concentration in supernatants in MH-7A cells (Fig. 2d).

### JNK inhibitor (SP600125) has anti-oxidative and anti-inflammatory effects in MH7A cells

**Fig. 3:**
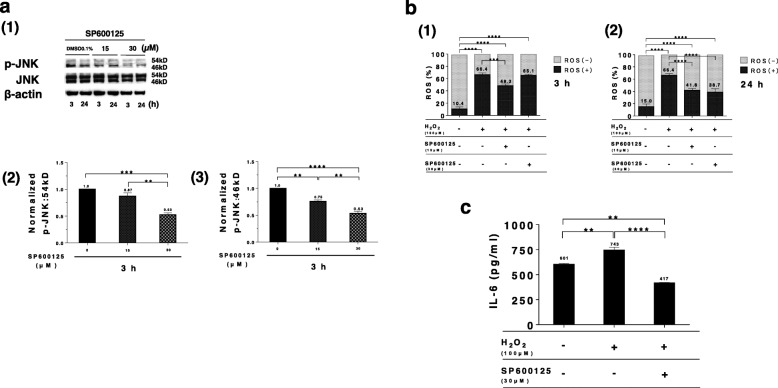
JNK inhibitor (SP600125) has anti-oxidative and anti-inflammatory effects. **a**(1) An illustrative Western blot picture of phosphorylated JNK (54 and 46 kD) in MH7A cells following SP600125 treatment (15 and 30 μM) for 3 h. **a**(2, 3) Normalized intensities were graphed (*N* = 3). Normalization was performed with phosphorylated JNK (54 and 46 kD) of untreated MH7A cell (3-h incubation). **b**(1, 2) ROS formation assay in MH7A cells by H_2_O_2_ treatment (100 μM) for 1 h following SP600125 treatment (15 and 30 μM) for 3 and 24 h was evaluated by Muse Cell Analyzer. **c** This graph showed the mean concentrations of IL-6 in supernatant of MH7A cells determined by chemiluminescent enzyme immunoassay (CLEIA) following 24 h after the H_2_O_2_ treatment (100 μM) and SP600125 (30 μM). All values are expressed as the mean ± SEM (*N* = 3). The significance levels are shown as *P value < .05, **P value < .01, ***P value < .001, ****P value < .0001

JNK inhibitor (SP600125) concentrations of 15 and 30 μM were selected based on the previous research [19, 20]. SP600125 was dissolved in DMSO and used at a final concentration of 0.1% or less. Phosphorylation of JNK (54 kD) expression showed dose-dependent decrease by SP600125 treatment. The intensity of band expression of p-JNK (54kD) under 3 h treatment of 30 μM SP600125 was significantly decreased than those of untreated and 15 μM SP600125 (0.53 vs. 0.87, *P* = .0026 and 0.53 vs. 1.0, *P* = 0.0005, respectively) (Fig. 3a(1, 2)). Phosphorylation of JNK (46 kD) expression also showed dose-dependent decrease by SP600125 treatment. The intensity of band expression of p-JNK (46kD) under 3 h treatment of 30 μM SP600125 was significantly decreased than those of untreated and 15 μM SP600125 (0.53 vs. 0.75, *P* = .004 and 0.53 vs. 1.0, *P* < .0001, respectively) (Fig. 3a(1, 3)). In addition, the intensity of band pattern of p-JNK (46kD) under 3 h treatment of 15 μM SP600125 was also significantly decreased than that of untreated condition (0.75 vs. 1.0, *P* = 0.0025) (Fig. 3a(1, 3)).

There was no significant difference in phosphorylation of JNK (54 and 46 kD) at 24 h compared with 0.1% DMSO-treated cells (Fig. 3a(1)).

Next, we measured the rate of ROS positive cells to clarify whether JNK inhibitor has an antioxidative effect like NAC.

Compared to untreated MH7A cells, 1 h treatment of H_2_O_2_ (100 μM) significantly increased ROS to 66.4% (*P* < .0001). SP600125 (15 μM) administration for 3 h significantly decreased H_2_O_2_-induced ROS to 48.3% (*P* < .0005). However, SP600125 (30 μM) administration for 3 h did not significantly decrease H_2_O_2_-induced ROS (Fig. 3b(1)).

Compared to untreated MH7A cells, 1 h treatment of H_2_O_2_ (100 μM) significantly increased ROS to 66.4% (*P* < .0001). SP600125 (15 μM) administration for 24 h significantly decreased H_2_O_2_-induced ROS to 41.6% (*P* < .0001). SP600125 (30 μM) administration for 24 h also significantly decrease H_2_O_2_-induced ROS to 38.7% (*P* < .0001) (Fig. 3b(2)).

These findings were confirmed by Western blotting of JNK proteins. Under identical conditions, phosphorylation of JNK (46 kD) in MH7A cells increased significantly 1 h after administering H_2_O_2_ (100 μM). However, phosphorylation of JNK decreased significantly 1 h after the administering of H_2_O_2_ (100 μM) and 3 and 24 h after administering SP600125 (15 or 30 μM) (Supplementary information: Fig. S1).

SP600125 treatment (30 μM) for 24 h significantly decreased IL-6 concentration compared with the condition under H_2_O_2_ (100 μM) for 24 h without SP600125 (417 vs. 743 pg/ml, *P* < .0001).

These findings indicated that SP100625 was able to reduce IL-6 concentration in supernatants in MH-7A cells (Fig. 3c).

### JNK inhibitor (SP600125) suppresses MMP-3 in both MH7A cells and RA-FLS

**Fig. 4:**
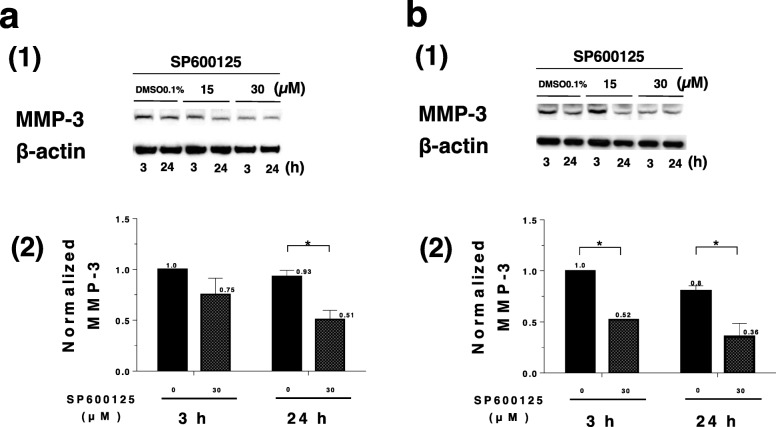
JNK inhibitor suppresses MMP-3 in both MH7A cells and RA-FLS. **a**(1) An illustrative Western blot picture of MMP-3 following SP600125 treatment (15 and 30 μM) after 3 and 24 h in MH7A cells. **a**(2) Normalized intensities were graphed (*N* = 3). Normalization was performed with MMP-3 band intensity of untreated MH7A cell (3-h incubation). All values are expressed as the mean ± SEM (*N* = 3). The significance levels are shown as *P value < .05. **b**(1) An illustrative Western blot picture of MMP-3 following SP600125 treatment (15 and 30 μM) after 3 and 24 h in RA-FLS. **b**(2) Normalized intensities were graphed (*N* = 3). Normalization was performed with MMP-3 bad intensity of untreated RA-FLS cell (3-h incubation). All values are expressed as the mean ± SEM (*N* = 3). The significance levels are shown as *P value < .05

In MH7A cells, protein expression of MMP-3 showed a dose-dependent decrease at 3 and 24 h. SP600125 (30 μM) treatment for 24 h significantly decreased MMP-3 expression compared with untreated cells (0.51 vs. 0.93, *P* = .0457), but not in the condition of SP600125 (30 μM) treatment for 3 h (Fig. 4a(1, 2)).

In RA-FLS, protein expression of MMP-3 showed a dose-dependent decrease at 3 and 24 h.

SP600125 (30 μM) treatment for both 3 and 24 h significantly decreased MMP-3 expression compared with untreated cells (0.52 vs. 1.0, *P* = .0359 at 3 h and 0.36 vs. 0.8, *P* = .0042, respectively) (Fig. 4b(1, 2)).

## Discussion

We found that low doses of NAC (1000 μM) and SP600125 (15 and 30 μM) were effective in suppressing the production of the proteolytic enzyme MMP-3, which, through suppression of JNK pathway component phosphorylation, causes joint destruction. The concentration of NAC required to suppress all pro-inflammatory cytokines and NF-κB is at least 5 mM. We determined that after 24 h of treatment, 10 mM NAC is cytotoxic to MH7A cells with only 30% of MH7A cells remaining viable. We utilized a 1000 μM concentration of NAC, which was cytotoxic to 10% or less of the MH7A cells in our sample. Low-dose NAC can reduce H_2_O_2_-induced ROS and increases in Nrf2 and p62 expression, which have antioxidant effects, and induce antioxidant genes (Fig. 1b, c, d) [21–26]. The expression of NF-κB is related to IL-6 production. Fujisawa et al. reported that low-dose NAC (1000 μM) is unable to suppress the transcriptional activity of NF-κB, which was consistent with our findings (Fig. 2c, d) [10]. However, we found that low-dose NAC (1000 μM) could significantly suppress the phosphorylation of JNK and downstream MMP-3 protein expression (Fig. 2a, b). These findings confirm that low-dose NAC is linked to the suppression of JNK phosphorylation and MMP-3 expression.

We conducted experiments using SP600125, a JNK inhibitor, to confirm that the suppression of JNK was directly inhibited MMP-3 expression. In MH7A cells, phosphorylation of JNK pathway components was significantly suppressed, dependent on dose, 3 h after SP600125 treatment (15 and 30 μM), and MMP-3 expression was significantly suppressed 24 h after administering SP600125 (30 μM; Figs. 3a and 4a). ROS, which significantly increased 1 h after administering H_2_O_2_ (100 μM), was significantly suppressed 3 h after SP600125 treatment (15 μM). At 24 h after administering SP600125 (15 and 30 μM), ROS was also significantly suppressed (Fig. 3b). The production of IL-6 in the cell supernatant was significantly inhibited 24 h after SP600125 treatment (30 μM; Fig. 3c).

Findings acquired in our study demonstrated that low-dose NAC suppressed MMP-3 and ROS, but did not inhibit IL-6 and that SP600125 suppressed all the three MMP-3, ROS, and IL-6 in MH-7A cells. In particular, we confirmed that SP600125 (30 μM) had an antioxidant, anti-inflammatory effect that suppressed ROS and inhibited IL-6 production (Fig. 3b, c, and S1). Experiments using RA-FLS obtained through the primary dispersion culture of synoviocytes taken from RA patients found that MMP-3 was significantly suppressed at 3 and 24 h following the administering of SP600125 (30 μM; Fig. 4b), which was similar results in the case of MH7A cells. We believe that this indicates the presence of an MMP-3 suppression mechanism that utilizes the JNK pathway in RA-FLS.

RA treatment must control both joint inflammation and joint destruction [1–4, 8–10]. Shen H et al. indicated that JNK inhibitor (SP600125) suppressed the increase of activator protein-1 (AP-1) transcription factor unstream of MMPs production and the increase of NF-κB (p65) transcription factor unstream of IL-6 production due to paraquat injury in human lung basal epitherial calls. [27]. Using MH7A cells, RA synovial model cells, we demonstrated that a JNK inhibitor suppresses inflammation and joint destruction.

**Fig. 5:**
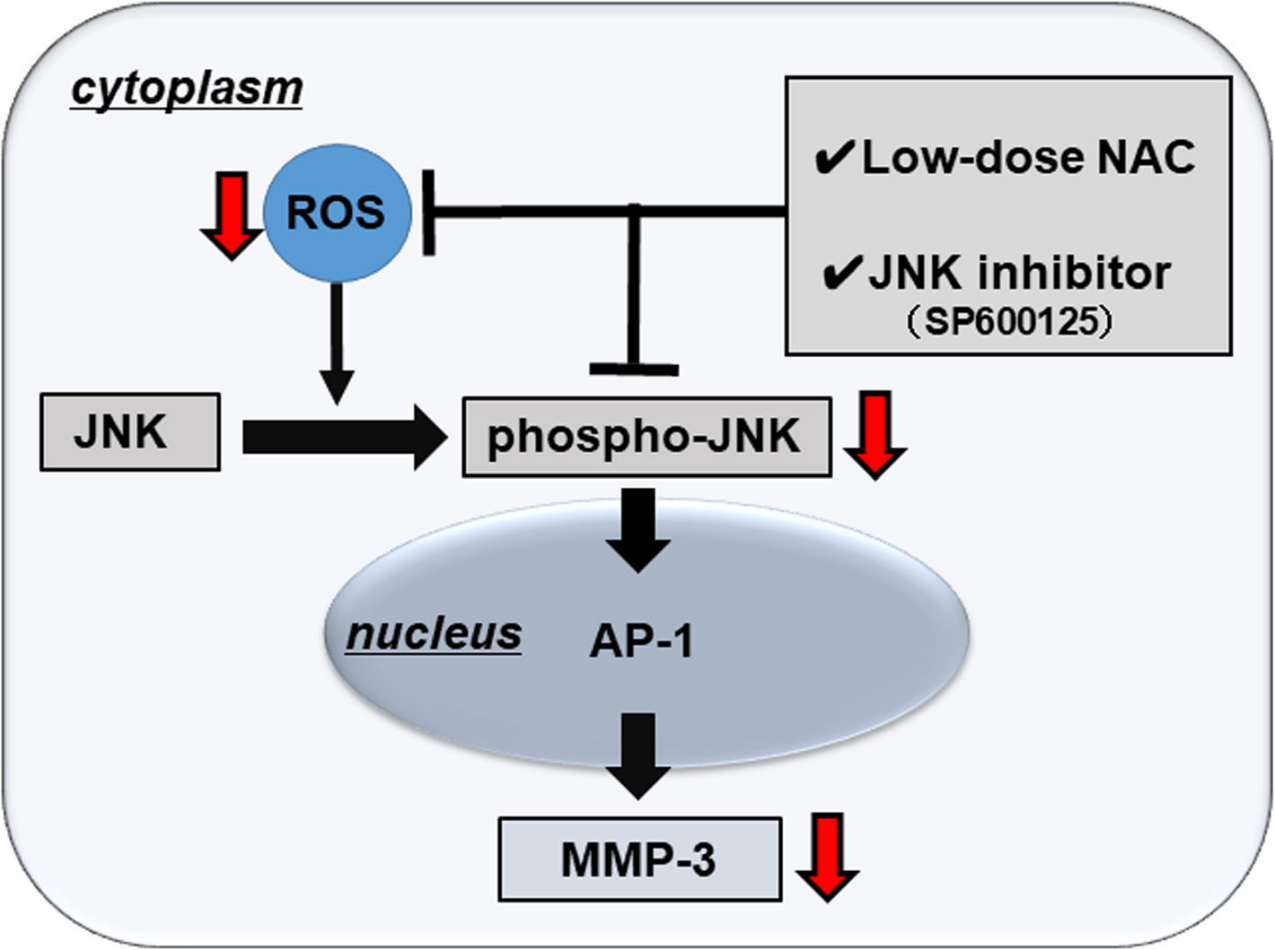
Schema of MMP-3 suppression via the JNK pathway by low-dose NAC and JNK inhibitor

We finally considered the explainable mechanism of MMP-3 suppression via JNK pathway by low-dose NAC and JNK inhibitor (Fig. 5). MMP-3 expression is regulated by JNK pathway. Once ROS activate JNK-pathway, phosphorylated JNK activates nuclear transcription factor AP-1 and MMP-3 protein production is promoted. Low-dose NAC or JNK inhibitor (SP600125) inhibits ROS production itself and specifically inhibits phosphorylation of JNK protein so MMP-3 protein production is prominently suppressed.

## Conclusions

MMP-3, which causes IL-6 production and joint destruction in RA patients, is produced by MH7A cells. In MH7A cells, NAC reduces ROS and MMP-3. A JNK inhibitor reduces ROS production, decreases IL-6, and downregulates MMP-3. MMP-3 is also reduced in human synoviocytes collected from RA patients following the JNK inhibitor treatment. We believe that JNK pathway will be a novel therapeutic target for the treatment of RA.

## Supplementary information

**Additional file 1: Fig. S1.** An illustrative Western blot picture of p-JNK and JNK by H_2_O_2_ treatment (100 μM) for 1 h following SP600125 treatment (15 and 30 μM) for 3 or 24 h in MH7A cells. SP600125 (15 and 30 μM) suppressed phosphorylation of JNK that increased 1 h after administering of H_2_O_2_ (100μM).

## Data Availability

The datasets used during the current study are available from the corresponding author on reasonable request.

## Abbreviations

AP-1: Activator protein -1
BSA: Bovine serum albumin
CLEIA: Chemiluminescent enzyme immunoassay
DAPI: 4',6-Diamidino-2-phenylindole
DMARDs: Disease-modifying anti-rheumatic drugs
DMSO: Dimethyl sulfoxide
DPBS: Dulbecco’s phosphate-buffered saline
FBS: Fetal bovine serum
JNK: c-Jun N terminal kinase
MAPK: Mitogen-activated kinase
MMPs: Matrix metalloproteinases
NAC: *N*-acetylcysteine
NF-κB: nuclear factor-kappa B
Nrf2: Nuclear factor erythroid 2-related factor 2
NSAIDs: Nonsteroidal anti-inflammatory drugs
OD: Optical density
p62: Sequestosome 1
PBS: Phosphate-buffered saline
RA: Rheumatoid arthritis
RA-FLS: Rheumatoid arthritis-fibroblast-like synoviocytes
ROS: Reactive oxygen species
SDS-PAGE: Sodium dodecyl sulfate-polyacrylamide gel electrophoresis
SEM: Standard error of mean
TBS: Tris-buffered saline
