# Supplementary figures and images for: The JNK pathway represents a novel target in the treatment of rheumatoid arthritis through the suppression of MMP-3
Source: J Orthop Surg Res. 2020 Jul 17;15:87. doi: 10.1186/s13018-020-01595-9 (PMC7371465; doi:10.1186/s13018-020-01595-9)

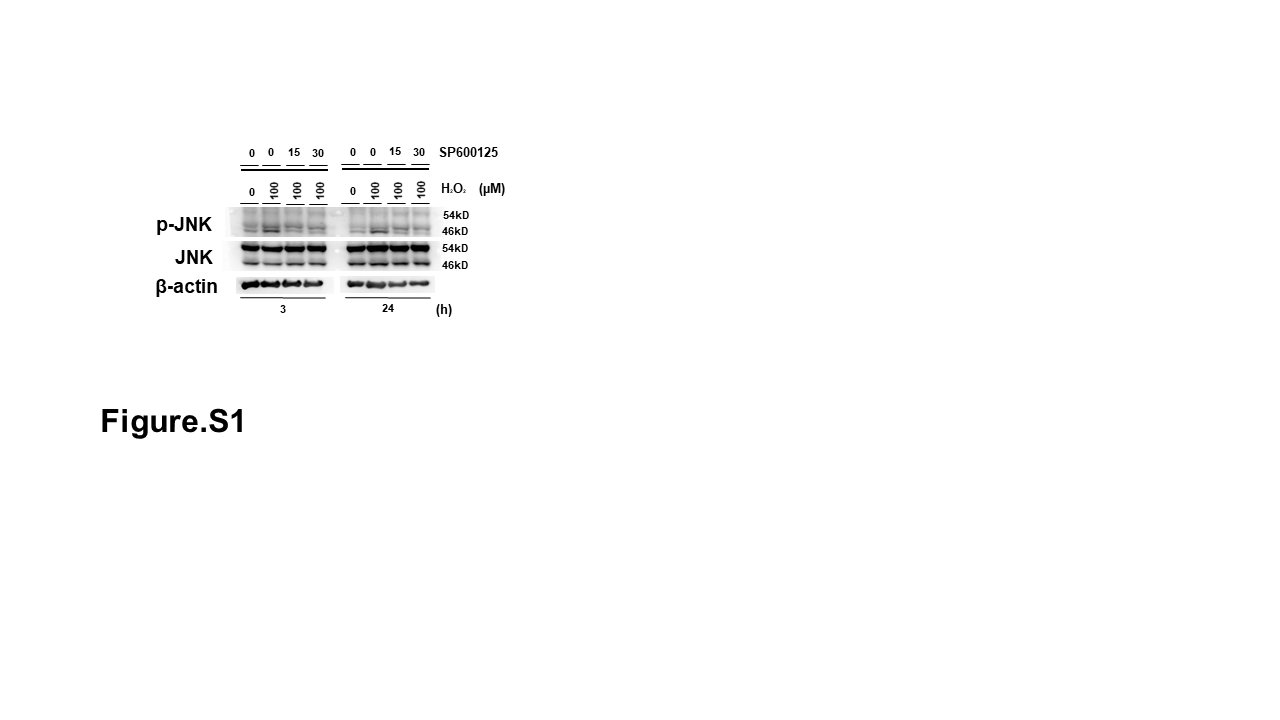

Supplement: Supplementary file 1 — Additional file 1: Fig. S1. An illustrative Western blot picture of p-JNK and JNK by H2O2 treatment (100 μM) for 1 h following SP600125 treatment (15 and 30 μM) for 3 or 24 h in MH7A cells. SP600125 (15 and 30 μM) suppressed phosphorylation of JNK that increased 1 h after administering of H2O2 (100μM). [file 13018_2020_1595_MOESM1_ESM.tif]
